# Does salinity affect lifestyle switching in the plant pathogen *Fusarium solani*?

**DOI:** 10.1099/acmi.0.000114

**Published:** 2020-03-16

**Authors:** Louise Eydoux, Emily C. Farrer

**Affiliations:** ^1^​ Department of Ecology and Evolutionary Biology, Tulane University, New Orleans, LA 70118, USA; ^2^​ Ecole Nationale Supérieure d’Agronomie de Toulouse, Avenue de l’Agrobiopole, 31326, Auzeville-Tolosane, France

**Keywords:** endophytes, mutualism, salinity stress, rice, *Oryza sativa*

## Abstract

Symbiotic microbes that live within plant hosts can exhibit a range in function from mutualistic to pathogenic, but the reason for this lifestyle switching remains largely unknown. Here we tested whether environmental stress, specifically salinity, is a factor that can trigger lifestyle switching in a fungus mainly known as a pathogen, *Fusarium solani. F. solani* was isolated from roots of *Phragmites australis* (common reed) in saline coastal marshes of Louisiana, USA, and we used *Oryza sativa* (rice) as a model organism from wetland environments to test the symbiont lifestyle. We plated rice seeds on control plates or plates with *F. solani* at three levels of salinity (0, 8 and 16 p.p.t.), then assessed germination and seedling growth after 20 days. Salinity strongly reduced percentage germination, slowed the timing of germination and reduced growth of rice. *F. solani* slowed germination, and it also caused a minor increase in root growth at medium salinity and a minor decrease in root growth at high salinity. Overall, despite being a common pathogen in other crop species (peas, beans, potatoes and many types of cucurbits), we found little evidence that *F. solani* has a strong pathogenic lifestyle in rice and we found weak evidence that pathogenicity may increase slightly with elevated salinity. These results have implications for both crops and native plant health in the future as soil salinization increases worldwide.

## Introduction

Symbiotic microbes that live within plant hosts are often classified as mutualistic, parasitic or pathogenic, but these strict categories belie the dynamic nature of plant–microbe interactions [1]. It is becoming increasingly recognized that microbes can exhibit a range in function from mutualistic to pathogenic/parasitic [2–4]; this change in the nature of the interaction is known as lifestyle switching. However, we still lack many case studies demonstrating lifestyle switching in different microbial taxa, and the underlying causes of lifestyle switching remain largely untested.

Environmental variability is one factor that has been found to cause a lifestyle switch in microbial symbionts. It is well known that mycorrhizal fungi often experience a switch from mutualistic at low soil nutrient levels to parasitic in fertilized soils [2]. The dark septate endophyte *Periconia macrospinos* also shifts from mutualistic to parasitic with increasing shade [4]. These switches in lifestyle are probably triggered by the nutritional costs and benefits of the fungi for the plant; when plants have ample nutrients or low carbon the benefits of the fungi do not outweigh the costs. In other cases, the lifestyle switching may not depend on plant nutritional status. For example, the mutualistic effect of the fungus *Curvularia* has been found to increase with thermal stress [5]. In addition, the fungus *Diplodia mutila* switches from symbiotic to pathogenic in high light conditions due to light-induced toxin production [6].

Here we test whether salinity can induce lifestyle switching in a fungus isolated from a saltmarsh in Louisiana, USA. Salinity stress is highly important to both natural and agricultural systems. In natural systems, in particular, salinity stress is predicted to increase in the future with sea-level rise and an increase in hurricanes and storms that cause saltwater intrusion into freshwater and brackish areas. Microbial symbionts are well known to promote salinity tolerance of the plant host [7–10]. On the other hand, weakened plant condition due to salinity stress may allow pathogens to express more virulent lifesyles [11]. We isolated the common pathogen *Fusarium solani* from asymptomatic *Phragmites australis* (common reed) plants from a Louisiana saltmarsh. *F. solani* is known for its pathogenicity in many crop systems including peas, beans, potatoes and curbutis [12, 13]. We use *Oryza sativa* (rice) as a model plant from wetland environments [7] to test the effect of salinity on symbiont lifestyle switching.

## Methods

### Isolation and identification of symbiont

The fungal symbiont was isolated from *Phragmites australis* (common reed) growing in a saltmarsh at the Louisiana Marine Consortium (LUMCON, 29.25° N 90.66° W), in the Louisiana Gulf coast. It was identified with Sanger sequencing of the ITS–LSU region with primers ITS1F/LR3 [14], with a 99.82 % match to *F. solani* in NCBI blast (GenBank accession number MN644607). The isolate was vouchered and archived in the Farrer Lab at Tulane University (culture collection accession number 203) and was regrown from voucher for this experiment.

The fungal isolate was plated on Malt Extract Agar (MEA - 10g of Difco Malt Extract, 10g of Fisherbrand Agar Powder and 500mL of DI water) and incubated at 26 °C for 10 days. Then it was plated on five plates each of MEA agar amended with a certain amount of NaCl (0 as low salinity, 8 g l^–1^ as medium salinity, 16 g l^–1^ as high salinity) (15 plates in total). We chose these salinities because they represent realistic conditions in coastal marshes: low represents freshwater marsh (0 p.p.t.), medium represents brackish marsh (8 p.p.t.) and high represents saltmarsh (16 p.p.t.) [15]. These cultures were allowed to grow for 20 days until the fungus covered the plate.

### Symbiont inoculation and plant growth

Rice (*O. sativa* L., variety Rex, purchased from Seed Ranch) was chosen as a model organism from wetland systems that represents how the host *Phragmites australis* may respond to inoculation. *Phragmites australis* seed from populations in Louisiana are often sterile (E. C. Farrer, unpublished data), and it is difficult to remove their native seed-borne microbes [16]. *Phragmites australis* and rice have similar responses in extreme conditions (including against pollutants and anoxic conditions) [17, 18], and rice has been used as a model organism in other studies testing the microbial effects on *Phragmites australis* [16], making rice an appropriate model organism in this case.

To surface-sterilize rice seeds, they were soaked in an 95 % ethanol solution for 3 min, dipped in 0.825 % sodium hypochlorite solution for 30 min and then rinsed with sterile water. More stringent sterilization procedures resulted in seed inviability. Some seeds were pressed on MEA agar and incubated (26 °C) for 1 week. As nothing grew, it confirmed the lack of fungi and bacteria on the surface of seeds.

To inoculate rice seeds with the symbiont, nine surface-sterilized seeds were placed on each culture plate containing the fungus (45 seeds per salinity level, 135 seeds in total). As a control, nine surface-sterilized seeds were placed on plates without the fungus as well (45 seeds per salinty level, 135 seeds in total) [19]. Plates were then placed in a growth chamber (40 % humidity, 12 h daily light exposure, 24 °C). Each day, seeds were checked and germination rates were documented. They were left to grow for a period of 20 days. Plants *in vitro* were then collected and the lengths of the stem and the longest root were recorded.

While seeds were successfully surface-sterilized (as shown above), the experiment found that they actually contained a seed endophyte. An unidentified fungus was present growing on the control (non-*Fusarium*) plates at the end of the experiment. As this seed endophyte was present in all seeds (all seeds in the control plates had colonies growing near them), we do not believe this detracts from our experimental results because all seeds had the same starting conditions prior to plating (see Discussion).

To determine the effect of *Fusarium* on germination, we calculated two variables: germination after 20 days and time to 10 % germination. We used a logistic regression (function glm) with a type III ANOVA in R [20] to test the effect of the presence of *Fusarium,* salinity level and their interaction on germination after 20 days. We used a linear model accounting for heterogeneous variances (function gls in package nlme) with type III ANOVA to test the effect of *Fusarium,* salinity level and their interaction on time to 10 % germination.

To test the effect of *Fusarium*, salinity and their interaction on stem and root length, we used linear models accounting for heterogeneous variances (function gls) and a type III ANOVA. We then used post-hoc tests in the multcomp package to determine which treatments were significantly different from one another.

## Results

### Germination

**Fig. 1.**
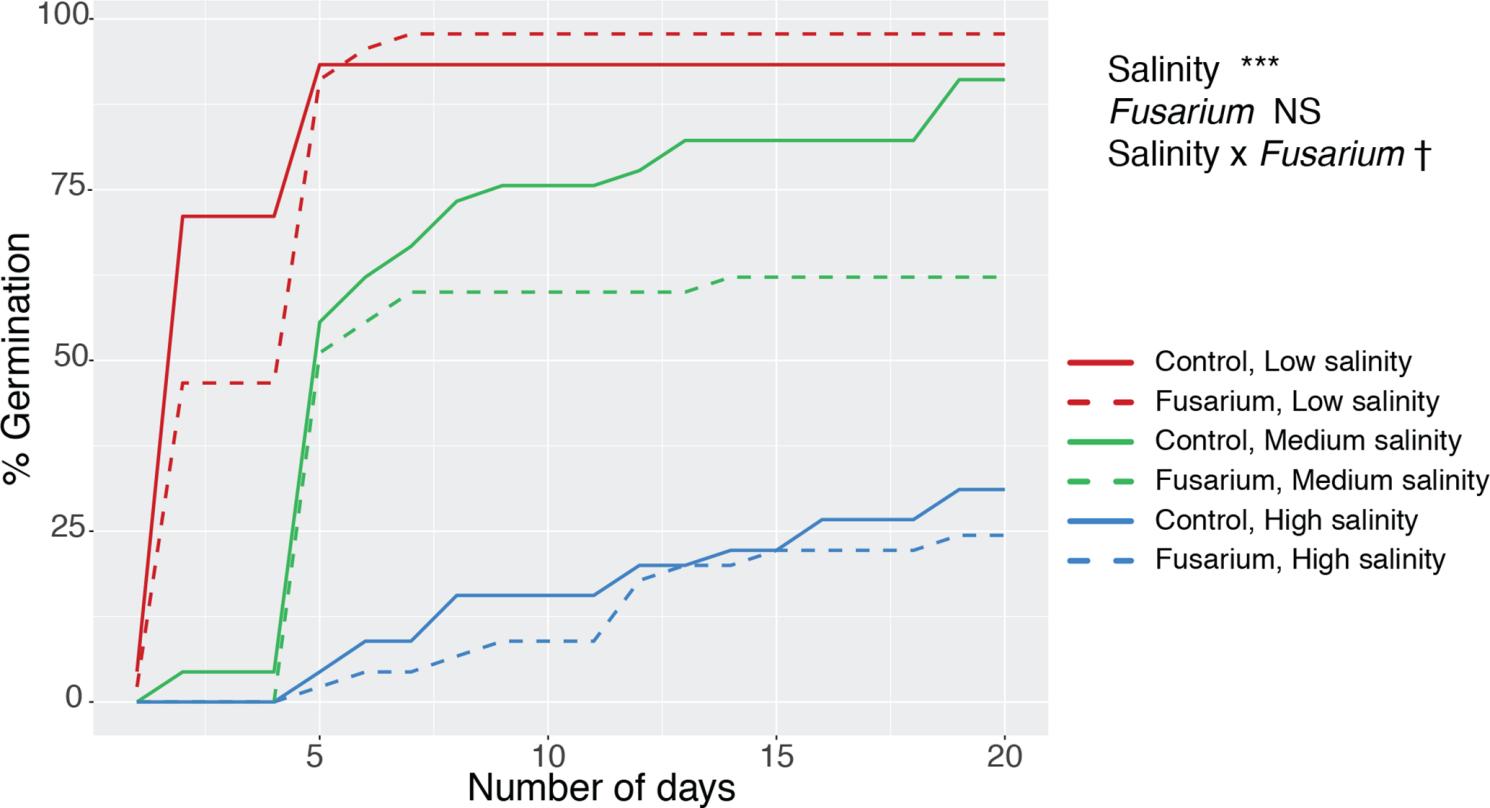
Germination over time at low (blue), medium (green) and high (red) salinity, without *Fusarium* (solid lines) and with *Fusarium* (dashed lines). We used 270 seeds in total, 45 per line (nine seeds per plate, five plates per line). Statistical results shown are from a logistic regression: †*P*<0.1, *P<0.05, **P<0.01, ***P<0.001, NS non-significant.

Salinity strongly reduced germination, with 31.1 % germination at high salinity versus 91.1 % at medium and 93.3 % at low salinity (Fig. 1, χ^2^=105.1, *P*<0.001). There was a nearly significant trend that *Fusarium* affected per cent germination differently in the different salinity treatments, suggesting lifestyle switching (χ^2^=6.0, *P*=0.0503): *Fusarium* reduced per cent germination most at medium salinity (Fig. 1).

**Fig. 2.**
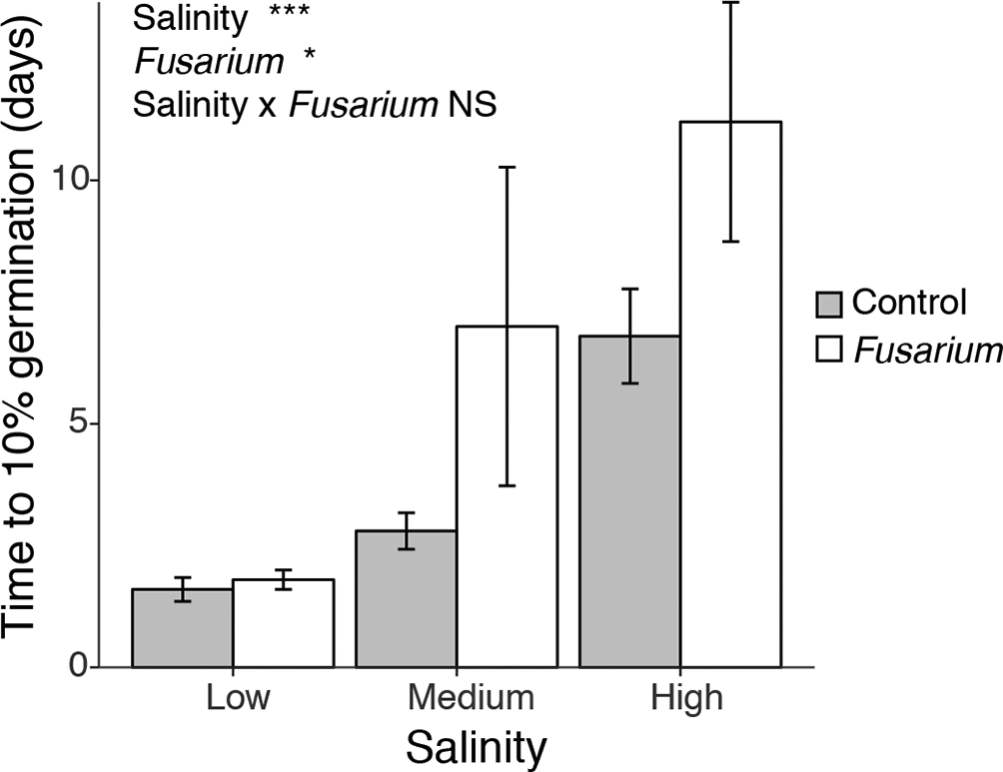
The effect of salinity and *Fusarium solani* on the time (days) it took for 10 % of the seeds to germinate. Error bars represent +/- 1 standard error of the mean. Statistical results shown are from an ANOVA: †*P*<0.1, *P<0.05, **P<0.01, ***P<0.001, NS non-significant.

Seeds at higher salinity germinated more slowly (Fig. 2, *F*_2,24_=16.8, *P*<0.001), and the presence of *Fusarium* also slowed the speed of germination (Fig. 2, *F*_1,24_=4.32, *P*=0.049), but there was no evidence of lifestyle switching.

### Plant growth

**Fig. 3.**
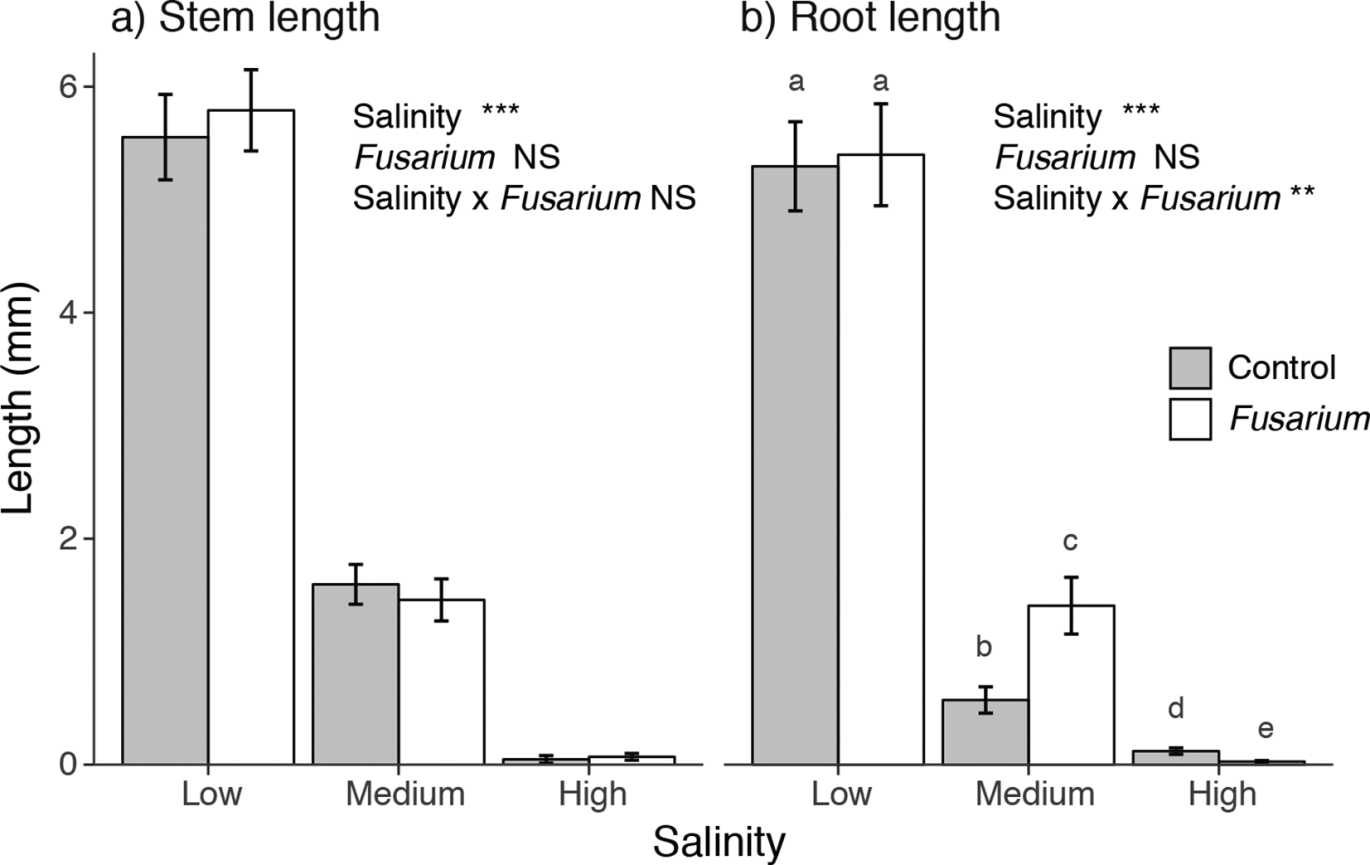
The effect of salinity and *Fusarium solani* on seedling stem length (a) and root length (b). Error bars represent +/- 1 standard error of the mean. Statistical results shown are from ANOVAs: †*P*<0.1, *P<0.05, **P<0.01, ***P<0.001, NS non-significant. Letters above the bars indicate significant differences among treatments based on a Tukey post hoc test (alpha=0.05).

Salinity had strong negative effects on stem length (*F*_2,264_=290.2, *P*<0.001) and root length (*F*_2,251_=173.2, *P*<0.001) (Fig. 3). *Fusarium* did not significantly affect stem length, but for root length the effect of *Fusarium* depended on salinity level, indicating lifestyle switching (significant *Fusarium* × salinity interaction, *F*_2,251_=4.86, *P*=0.009): *Fusarium* had a positive effect on root length at medium salinity and a negative effect at high salinity (Fig. 3b).

## Discussion

This study reports on the effect of salinity on the lifestyle of *F. solani*, a fungus mainly known as a plant pathogen, which was isolated from the roots of a wetland plant (*Phragmites australis*) in Louisiana. Seeds of *O. sativa*, a model wetland plant, were inoculated with this fungus. We show that, surprisingly, *F. solani* does not seem to have strong pathogenic effects across differents levels of salinity. It does slow germination, and it shows weak evidence of lifestyle switching, such that it is a weak mutualist promoting root growth at medium salinity, and a weak pathogen at high salinity.

### Effect of salinity on germination and growth of rice seeds

Salinity is a well-known stressor of plants. The ionic and osmotic stress as well as the associated reduction in water and nutrient availability can result in stunted growth and reduced yield [21] and inhibition of root development and germination of seeds [22–24]. Therefore, it is not surprising that, in our experiment, salinity reduced germination by 67 %, slowed time to germination, and strongly reduced both stem and root growth. Because soil salinzation is currently affecting large tracts of both natural and agricultural areas and its impacts are projected to increase steeply in the future [25–27], it is important to assess whether microbial symbionts may ameliorate or exacerbate these detrimental effects of salinity.

### Effect of *Fusarium* on germination and growth and evidence for lifestyle switching

Despite the fact that *F. solani* is known be a virulent pathogen in many crop species [28, 29], we found little evidence that it was highly pathogenic in rice. The only consistent negative effect it had on rice across all salinity treatments was that it slowed the germination rate of rice seeds. While *F. solani* has been isolated from a large number of grasses, it has been suggested that it is not pathogenic in grasses [30]; however, it has been shown to be highly virulant to ryegrass (*Lolium*) [31, 32].

We detected weak evidence of lifestyle switching in *F. solani* in rice. *F. solani* had no effect on root growth at low salinity, promoted root growth under conditions of medium salinity and inhibited growth at high salinity. There was also a nearly significant trend that it reduced germination rate at medium salinity, but not at low or high salinity. Endophytes, including *Fusarium* spp., have been found to confer salinity tolerance to rice plants grown under moderate to high salinity [33]. Furthermore, consistent with our results, one *Fusarium* species has been shown to be most pathogenic to maize under stressful conditions [11], suggesting that high-salinity conditions may trigger pathogenicity of *F. solani* in rice.

### Limitations

One important limitation of this study was that while the rice seeds used were surface-sterilized, they were found to contain an endophyte. The endophyte was observed growing out of all rice seeds plated on control (non-*Fusarium*) plates. It was not observed growing in the *Fusarium* plates, probably because *Fusarium* itself was so dense on those plates, but we assume that the seeds contained the endophyte because it was so prevalent in the control plates and because all seeds came from the same batch. We do not think that the presence of the endophyte detracts from our experiment, because seeds in both treatments were infected with the endophyte and thus were consistent. Because this endophyte was naturally present in the seeds, our study actually might be quite biologically relevant in terms of testing how *F. solani* affects rice plants that are typically used for food production (which are not endophyte-free). However, this means that our ‘controls’ measure the germination and growth of rice with the endophyte, and our ‘*Fusarium*’ treatment represents germination and growth with both the endophyte and *Fusarium*. This result can be used to inform future work with commercial rice seeds: methods to remove seed endophytes, such as soaking seeds in fungicide, should be applied.

In conclusion, our study shows that salinity stress can cause lifestyle switching of *F. solani* in rice: it ranged from having no effect at low salinity, a positive growth effect at medium salinity, to a negative effect at high salinity. As endophytes play a key role for stress adaptation of plants in both natural and agricultural systems [8–10, 34], it is critical to understand which environmental conditions may trigger an endophytic lifestyle switch. We recommend that future research focuses on understanding the effect of salinity on lifestyle switching in *F. solani* in terrestrial crops, as their responses may be different from those observed here. Furthermore, endophytes are one potential tool that can be developed to promote plant growth and stress tolerance in soil that is increasingly affected by salinization [8, 33], and thus more research is necessary to understand how their effects on plants might shift over salinity gradients.
